# Endothelial and platelet dysfunction in systemic sclerosis with pulmonary hypertension

**DOI:** 10.1371/journal.pone.0357987

**Published:** 2026-09-16

**Authors:** Thamonwan Imerbtham, Thanaporn Sriwantana, Pintip Ngamjanyaporn, Thanyaluck Sotananusak, Pipat Ngammisri, Krittimeth Trerayapiwat, Panu Dumrongkitchaiporn, Prapaporn Pisitkun, Sirada Srihirun, Pornpun Vivithanaporn, Nathawut Sibmooh

**Affiliations:** 1 Program in Translational Medicine, Faculty of Medicine Ramathibodi Hospital, Mahidol University, Bangkok, Thailand; 2 Department of Cardio-Thoracic Technology, Faculty of Allied Health Sciences, Naresuan University, Phitsanulok, Thailand; 3 Chakri Naruebodindra Medical Institute, Faculty of Medicine Ramathibodi Hospital, Mahidol University, Samut Prakan, Thailand; 4 Division of Allergy Immunology and Rheumatology, Department of Medicine, Faculty of Medicine Ramathibodi Hospital, Mahidol University, Bangkok, Thailand; 5 Department of Pharmacology, Faculty of Dentistry, Mahidol University, Bangkok, Thailand; National Institutes of Health, National institute of Diabetes and Digestive and Kidney Diseases, UNITED STATES OF AMERICA

## Abstract

Systemic sclerosis (SSc) is an autoimmune connective tissue disease characterized by vasculopathy, inflammation, and fibrosis, in which pulmonary hypertension (PH) is a major cause of mortality. In SSc, the relationship between endothelial and platelet functions with echocardiographic findings have not been evaluated. Here, we evaluated the endothelial and platelet functions in SSc patients with different PH probability and tested their associations with echocardiographic parameters, including the maximal tricuspid regurgitant velocity (TRV_max_). Endothelial function was assessed by flow-mediated dilation (FMD) of brachial artery and blood nitrite levels, measured by chemiluminescence method. Platelet activity was measured by light transmission aggregometry in response to stimulation by adenosine diphosphate (ADP) and thrombin receptor–activating peptide-6 (TRAP-6). PH probability was assessed by echocardiography, and PH diagnosis was confirmed by right heart catheterization. SSc patients with PH probability had lower FMD than age-matched controls: FMD decreased from 13.3 ± 3.9% in controls to 5.6 ± 1.7%, 4.6 ± 0.4%, and 3.8 ± 0.4% in SSc patients with low PH probability, intermediate PH probability, and confirmed PH, respectively. SSc patients with PH had higher blood nitrite levels than controls or the patients with low PH probability. TRV_max_ correlated inversely with FMD and positively with platelet aggregation. In multivariable regression analysis, TRAP-6-induced platelet aggregation and FMD were independently associated with TRV_max_. Furthermore, we demonstrated in two patients that nebulized sodium nitrite transiently reduced TRV_max_. In conclusion, SSc patients have endothelial dysfunction and increased platelet activity, which exhibits correlation with TRV_max_.

## Introduction

SSc is a chronic autoimmune connective tissue disease characterized by immune dysregulation, vasculopathy secondary to endothelial dysfunction, and progressive fibrosis of the skin and visceral organs, including the heart and lungs [1]. Pulmonary and cardiac complications are the leading causes of morbidity and mortality in SSc. PH is a serious complication that worsens the prognosis of SSc patients. SSc-related PH (SSc-PH) has poorer prognosis and treatment outcome than idiopathic PH [2]. Understanding the pathogenesis and development of an assessment tool to monitor SSc-PH progression are urgently required to change the course of the disease.

Vasculopathy and endothelial dysfunction are key characteristics of SSc and play a vital role in PH development. The endothelium regulates vascular tone, inflammation, thrombosis, and smooth muscle proliferation by synthesizing endogenous vasodilators and platelet inhibitors, including nitric oxide (NO). In SSc, the endothelial dysfunction can lead to abnormal vasoconstriction, inflammation, and vascular remodeling. Even in the absence of PH, the SSc patients had lower FMD than healthy subjects [3,4]. SSc patients have elevated serum or plasma NO, measured as total nitrate and nitrite (NO_x_) [5–7]. Yet, the specific levels of nitrite, a bioactive product of NO, or its association with SSc-PH has not been reported.

As endothelial dysfunction is a key characteristic contributing to pathogenesis of PH, in this study we investigated the endothelial function in SSc patients, measured as FMD, blood nitrite, and platelet activity, and evaluated their association with echocardiographic findings.

## Methods

### Study setting and participants

This study obtained the ethical approval from the Ramathibodi Hospital Ethics Committee at Mahidol University (ID 2024/646) and all participants provided written informed consent, in accordance with the principles of the Declaration of Helsinki. The recruitment period was from February 16, 2023, to January 31, 2026. To ensure transparency, the study protocol was recorded in the Thai Clinical Trials Registry (TCTR20150518002).

**Fig 1:**
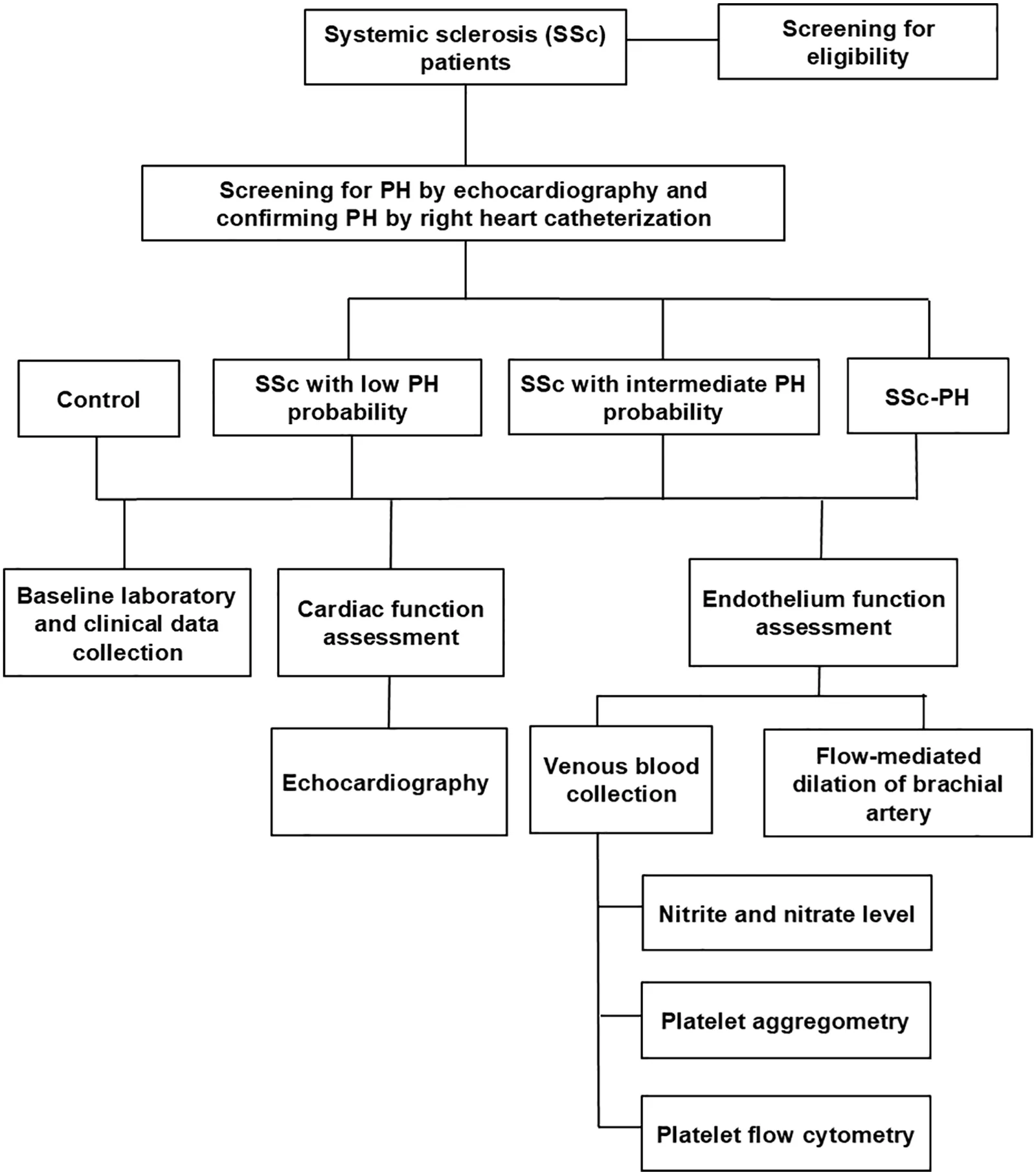
Study workflow. Clinically stable systemic sclerosis (SSc) patients and healthy controls were enrolled. SSc patients were categorized into low pulmonary hypertension (PH) probability, intermediate PH probability, and confirmed PH.

The study involved SSc patients who were clinically stable, along with age-matched healthy controls (Fig 1). According to the 2022 ESC/ERS guidelines [8], SSc patients were prospectively categorized into three groups according to their PH probability. All SSc patients underwent echocardiographic for screening of PH. By echocardiography, PH was suggestive when TRV_max_ > 3.4 m/s or a TRV_max_ of 2.9 to 3.4 m/s accompanied by at least two additional echocardiographic signs of PH which included right ventricular enlargement (right ventricular/left ventricular basal diameter ratio >1.0), interventricular septal flattening (D-shaped left ventricle), main pulmonary artery diameter >25 mm, right ventricular outflow tract acceleration time <105 ms (when assessable), early diastolic pulmonary regurgitation velocity >2.2 m/s (when assessable), right atrial area >18 cm², and inferior vena cava diameter >21 mm with reduced inspiratory collapse. These additional parameters were evaluated during standard 2D transthoracic echocardiography. If the patients had the echocardiographic criteria for PH, PH was ultimately confirmed by right heart catheterization (RHC) demonstrating a mean pulmonary arterial pressure (mPAP) over 20 mmHg at rest. Intermediate probability for PH was defined when TRV_max_ of 2.9 to 3.4 m/s without additional echocardiographic signs, or a TRV_max_  ≤ 2.8 m/s accompanied by at least two echocardiographic signs of PH. Low probability for PH was assigned to patient with a TRV_max_ ≤ 2.8 m/s and no additional echocardiographic features suggestive of PH.

The exclusion criteria included patients with major comorbidities, including heart failure, ischemic heart disease, chronic obstructive pulmonary disease, chronic kidney disease, active infection, malignancy, and NO donor use. Nonsteroidal anti-inflammatory drugs or agents affecting platelet function were discontinued for at least two weeks before the study.

### Baseline characteristics and laboratory parameters

Demographic and clinical data, including age, sex, body weight, height, body mass index (BMI), disease duration, comorbidities, and current medications, were obtained through structured interviews and review of electronic medical records. The clinical laboratory investigations; for example, complete blood count, creatinine, bilirubin, and lipids were performed at the Pathology Laboratory, Faculty of Medicine Ramathibodi Hospital. All the patients had undergone spirometry and the six-minute walk distance (6MWD) test as the guidelines [9,10]. High-resolution computed tomography (HRCT) of chest was used to assess interstitial lung disease.

### FMD of the right brachial artery

FMD was assessed using a portable ultrasound system (UProbe-C5D, Universal Diagnostic Solutions, Vista, CA, USA) [11]. Participants fasted overnight (10–12 h) and rested supine for approximately 20 minutes in a quiet, temperature-controlled room before image acquisition. The brachial artery was imaged longitudinally above the antecubital fossa, and baseline brachial artery diameter was recorded continuously for 30 seconds before cuff inflation. A blood pressure cuff was positioned on the distal forearm immediately below the medial epicondyle to minimize direct mechanical effects on the imaged brachial artery. The cuff was inflated to 50 mmHg above systolic blood pressure for 5 minutes and then rapidly deflated to induce reactive hyperemia. Continuous brachial artery imaging was recorded for up to 180 seconds after cuff release, and the highest 5-second average diameter was used as the peak diameter. FMD was calculated as the percentage change from baseline to peak brachial artery diameter.

To assess intra-observer reproducibility, FMD measurements from 10 randomly selected participants were reanalyzed by the same observer in a blinded manner. Agreement between repeated measurements was evaluated using the intraclass correlation coefficient (ICC) based on a two-way mixed-effects model with absolute agreement.

### Blood nitrite and nitrate

We collected venous blood via catheter using heparin (143 units/10 mL) as an anticoagulant. The whole blood samples were rapidly mixed with nitrite-stabilizing solution (composed of 0.8 M ferricyanide, 10 mM N-ethylmaleimide, and 1% NP-40 in a 4:1 v/v ratio of sample to stabilizing solution) [12], and stored at −80 °C. Nitrite (NO_2_^-^) was measured using a tri-iodide-based chemiluminescence NO analyzer (CLD88; Eco Medics AG, Duernten, Switzerland). Plasma nitrate (NO_3_^-^) was measured by vanadium (III)-based chemiluminescence [13].

### Fractional exhaled NO (FE_NO_)

Exhaled breath samples were obtained at baseline from all participants utilizing an offline FE_NO_ collection apparatus linked to a reusable Mylar container (ECO MEDICS AG, Duernten, Switzerland). The Mylar container is impermeable and nonreactive to NO, thereby preserving sample integrity. NO concentrations were determined by interfacing the container with the inlet of a chemiluminescence NO analyzer, and findings were reported in parts per billion.

### Platelet aggregometry and flow cytometry

Venous blood was collected from the median cubital vein of fasting participants (10–12 fasting hours) using a 21-gauge winged needle into tubes containing 3.8% sodium citrate (9:1, blood-to-anticoagulant ratio) Platelet-rich plasma (PRP) was prepared by centrifugation at 200 × g for 10 min at 25°C, and platelet-poor plasma (PPP) was obtained by further centrifugation at 5,000 × g for 10 min. Platelet aggregation was performed within 2 hours of blood collection using a Chrono-Log aggregometer (Model 540 VS, Chrono-Log Corp., Havertown, PA, USA).

ADP is a weak platelet agonist released from platelet granules to amplify platelet activation. TRAP-6 stimulates platelets by activating thrombin receptor (protease-activated receptor 1), representing the initial trigger of platelet activation. PPP was used to calibrate 100% light transmission, and PRP was preincubated at 37°C before stimulation with ADP (2 and 10 μM) or TRAP-6 (3 and 9 μM). Agonist concentrations were selected based on our preliminary dose–response experiments in healthy volunteers, yielding 50% effective concentration (EC₅₀) values of 2.67 ± 0.38 μM for ADP and 8.00 ± 1.24 μM for TRAP-6 (S1 Fig). Therefore, ADP at 2 μM and TRAP-6 at 9 μM were selected as submaximal concentrations to maximize sensitivity for detecting differences in platelet reactivity.

For flow cytometry, freshly prepared PRP was diluted 1:10 and stained with FITC-conjugated PAC-1 (activated GPIIb/IIIa), PE-conjugated anti-CD62P (P-selectin), and PE-Cy5-conjugated anti-CD42b (platelet marker). Samples were incubated with phosphate-buffered saline or ADP (0.5 or 1 μM) for 15 min at room temperature in the dark, fixed with 1% paraformaldehyde for an additional 15 min, and analyzed using a BD Accuri C6 Plus flow cytometer (BD Biosciences, San Jose, CA, USA). Platelets were identified by forward- and side-scatter characteristics and CD42b expression. Activated platelets were quantified as the percentages of PAC-1-positive and CD62P-positive events among 10,000 CD42b-positive platelets.

### Transthoracic echocardiography

Standard 2D transthoracic echocardiography was performed for all patients according to the American Society of Echocardiography Guidelines [14–16]. All examinations were conducted by a single experienced observer. Patients were in the left lateral decubitus position after a 10-minute rest. Imaging was acquired using an Affiniti 70C ultrasound scanner (Philips Healthcare, Amsterdam, The Netherlands) with an S5-1 broadband sector phased-array transducer. Grayscale recordings were optimized to achieve a mean frame rate ≥50 frames/s.

The estimated systolic pulmonary artery pressure (sPAP) was reported as the estimated right ventricular systolic pressure (eRVSP), calculated using the modified Bernoulli equation: eRVSP = 4 × (TRV_max_)² + RAP [14]. The right atrial pressure (RAP) was estimated based on the diameter and collapsibility of the inferior vena cava. Diastolic pulmonary artery pressure (PADP) was calculated as PADP = 4(end-diastolic pulmonary regurgitation velocity)^2^ + RAP. The mPAP was estimated as $\text{mPAP}=\frac{\text{1}}{\text{3}}(\text{sPAP})+\frac{\text{2}}{\text{3}}(\text{PADP})$.

TAPSE (tricuspid annular plane systolic excursion) to sPAP ratio was calculated as an additional marker of right ventricular (RV)–pulmonary arterial (PA) coupling. TAPSE reflects longitudinal right ventricular systolic shortening, while sPAP represents right ventricular afterload. A lower TAPSE/sPAP ratio indicates impaired RV–PA coupling.

### Sodium nitrite inhalation

As inhaled sodium nitrite (15 and 40 mg) can decrease pulmonary pressure in thalassemia patients with PH [17,18], we tested the effects on pulmonary pressure of inhaled sodium nitrite in two patients as case report. The maximum tolerated dose in healthy subjects is 90 mg [19]. Sterile sodium nitrite solution for intravenous injection, manufactured by Queen Saovabha Memorial Institute, The Thai Red Cross Society, Thailand, was diluted with saline and administered via a Beurer IH 25/1 nebulizer (Beurer Medical, Ulm, Germany). Normal saline was used as a control.

Patient 1 with confirmed SSc-PH received 15 mg of sodium nitrite inhalation, and patient 2 with an intermediate PH probability received incremental doses of 15 mg followed by 30 mg. Each patient first received 15 minutes of saline nebulization, followed by a 15-minute rest, and then 15 minutes of sodium nitrite nebulization, with a 15-minute interval between doses. During inhalation, TRV_max_ was measured by echocardiography every one minute. Systolic blood pressure, diastolic blood pressure and heart rate were assessed using a non-invasive automated sphygmomanometer at baseline, every 5 minutes during inhalation, and at various time points after inhalation cessation. Methemoglobin levels were measured at baseline and 15 minutes after the end of inhalation. Blood nitrite and nitrate levels were measured at baseline, immediately after the end of saline and nitrite inhalation, and 15 minutes and 30 minutes after inhalation.

### Statistical analysis

Statistical analyses were performed using GraphPad Prism® version 10.0 (GraphPad Software, San Diego, CA, USA) and IBM SPSS Statistics for Windows, version 26.0 (IBM Corp., Armonk, NY, USA). Group comparisons were conducted by GraphPad Prism, and correlation analyses were performed in SPSS. Figures were generated by GraphPad Prism. Normality was assessed with the Shapiro–Wilk test. Continuous variables are presented as means ± SD or medians (interquartile range), as appropriate, and categorical variables as frequencies and percentages. Comparisons among groups were performed using one-way analysis of variance with Tukey’s post hoc test or the Kruskal–Wallis test with Dunn’s post hoc test, as appropriate. Categorical variables were compared using a chi-squared test. Correlations were assessed with Spearman’s rank correlation coefficient. Statistical significance was defined as a two-tailed *p*-value < 0.05.

Multiple linear regression using the enter method was performed to evaluate the independent associations between endothelial dysfunction and platelet activation with TRV_max_. Predictor variables were selected a priori based on the study's biological hypothesis. FMD- and TRAP-6-induced platelet aggregation were included because they represent the two principal mechanistic pathways investigated: endothelial dysfunction and platelet activation. Prior to model interpretation, regression assumptions were assessed by evaluating residual normality using histograms and normal probability (P–P) plots, homoscedasticity using standardized residual-versus-predicted value plots, multicollinearity using variance inflation factors (VIFs), and independence of residuals using the Durbin–Watson statistic. Model stability was further evaluated using bootstrap resampling (1,000 samples) with bias-corrected and accelerated 95% confidence intervals.

## Results

### Baseline characteristics and laboratory data

Thirty-nine participants were enrolled, including 12 healthy controls and 27 SSc patients (Table 1). SSc patients were stratified by echocardiographic probability of PH into low probability (n = 12), intermediate probability (n = 7), and confirmed SSc-associated PH (SSc-PH; n = 8). All participants were females.

**Table 1:** Baseline characteristics and laboratory variables of the study population.

Parameters	Control (n = 12)	SSc		
		Low PH probability (n = 12)	Intermediate PH probability (n = 7)	Confirmed PH (n = 8)
Age	55.9 ± 2.6	54.2 ± 8.3	55.1 ± 7.0	57.7 ± 7.2
Systolic BP, mmHg	123.1 ± 20.2	118.8 ± 14.6	121.9 ± 14.2	125.8 ± 15.4
Diastolic BP, mmHg	82.8 ± 4.6	76.7 ± 8.7	75.6 ± 7.0	72.2 ± 16.5
Heart rate, beats/minute	72.5 (66.0-75.7)	82.0 (74.0-90.7)	84.0 (73.0-103.0)	79.5 (75.7-88.2)
Body mass index, kg/m^2^	22.1 ± 2.5	21.1 ± 1.7	24.3 ± 3.4	19.6 ± 5.9
**Laboratory**				
White blood cells, x10^3^ /μL	5.7 (4.7-7.6)	6.3 (4.8-7.4)	7.2 (5.5-7.7)	6.4 (4.3-8.6)
Hemoglobin, g/dL	11.7 ± 0.8	10.6 ± 1.7	12.4 ± 0.4	11.9 ± 1.3
Platelets, x10^3^ /μL	281.3 ± 81.7	261.1 ± 82.4	277.2 ± 56.5	237.9 ± 63.1
Creatinine, mg/dL	0.7 ± 0.1	0.7 ± 0.2	0.8 ± 0.1	0.9 ± 0.3
AST, U/L	23.4 ± 6.3	26.6 ± 5.0	29.0 ± 3.1	24.6 ± 8.2
ALT, U/L	18.3 ± 6.5	19.2 ± 7.5	22.2 ± 7.5	15.0 ± 6.0
Triglyceride, mg/dL	96.4 ± 31.6	113.5 ± 34.3	94.6 ± 6.9	120.3 ± 29.4
Cholesterol, mg/dL	197.0 ± 23.4	172.8 ± 8.4	181.8 ± 22.7	203.4 ± 52.1
HDL, mg/dL	62.2 ± 10.4	54.8 ± 3.4	59.4 ± 3.6	66.4 ± 19.4
LDL, mg/dL	122.3 ± 8.1	112.7 ± 9.1	113.0 ± 11.5	119.3 ± 7.3

Values are means ± SD or medians (interquartile range).

Abbreviations: ALT, alanine aminotransferase; AST, aspartate aminotransferase; BP, blood pressure; HDL, high-density lipoprotein; LDL, low-density lipoprotein.

The patients had similar baseline characteristics, including age, blood pressure, and body mass index. There were no significant differences among the groups in hematological and biochemical laboratory values. Prothrombin time and international normalized ratio did not differ significantly among the low PH probability, intermediate PH probability, and SSc-PH groups (PT: 12.10 ± 0.26, 11.81 ± 1.42, and 12.48 ± 1.59 seconds, respectively, *p* = 0.74; INR: 1.03 ± 0.02, 1.00 ± 0.15, and 1.06 ± 0.10, respectively, *p* = 0.67).

### Patients’ clinical characteristics

All SSc patients were screened for PH by echocardiography. Of twenty-seven SSc patients, eight patients had suggestive findings of PH (TRV_max_ > 3.4 m/s or TRV_max_ of 2.9 to 3.4 m/s with at least two other echocardiographic signs of PH) and subsequently had undergone RHC. The diagnosis of PH was finally made by RHC in those patients who were then defined as SSc-PH. Their invasive hemodynamic parameters included mean pulmonary arterial pressure of 27.6 ± 7.9 mmHg, pulmonary arterial wedge pressure of 6.5 (5.0–11.0) mmHg, and pulmonary vascular resistance of 4.5 ± 2.3 Wood units. These parameters were consistent with pre-capillary pulmonary arterial hypertension. SSc-PH patients had a longer disease duration than those with low PH probability (Table 2).

**Table 2:** Clinical characteristics, pulmonary function test, and functional capacity of patients with systemic sclerosis.

Parameters	SSc with low PH probability (n = 12)	SSc with intermediate PH probability (n = 7)	SSc-PH (n = 8)
Disease duration, years	4.5 (3.0-6.7)	10.0 (3.0-17.0)	13.5 (7.0–16.2) ^a^
6MWD, meters	512.4 ± 57.5	432.7 ± 36.2 ^a^	404.1 ± 56.5 ^a^
Pulmonary fibrosis by HRCT, %	18.1 ± 7.6	18.2 ± 3.4	24.7 ± 8.4
FE_NO_, ppb	53.3 (37.0-90.5)	54.3 (36.0-62.2)	33.1 (31.1-46.3)
**SSc subtype, n**			
Diffuse	11	7	8
Limited	0	0	0
Sine SSc	1	0	0
**Medications, n**			
Prednisolone	7	6	8
Mycophenolate mofetil	8	7	8
Methotrexate	1	0	0
Azathioprine	2	0	0
Hydroxychloroquine	9	7	2
Nifedipine	9	7	5
Sildenafil	2	1	7
Bosentan	0	0	1
Aspirin	5	7	7
Statin	4	3	3
**Pulmonary function test**			
FVC, liters	2.3 ± 0.5	1.9 ± 0.4	1.7 ± 0.6
FEV_1_, liters	1.8 ± 0.5	1.6 ± 0.3	1.4 ± 0.5
FEV_1_/FVC	82.8 ± 7.7	81.4 ± 4.0	85.5 ± 5.8
TLC, liters	3.4 ± 0.5	3.0 ± 0.6	2.3 ± 0.3 ^a,b^
DLCO, mL/mmHg/min	10.9 ± 3.4	10.1 ± 2.3	7.9 ± 0.8 ^a^

6MWD, six-minute walk distance; DLCO, diffusing capacity of the lung for carbon monoxide; FE_NO_, fractional exhaled nitric oxide; FEV_1_, forced expiratory volume in one second; FVC, forced vital capacity; HRCT, high-resolution computed tomography; ILD, interstitial lung disease; PH, pulmonary hypertension; ppb, parts per billion; SSc, systemic sclerosis; TLC, total lung capacity

Values are presented as means ± standard deviation or medians (interquartile range), as appropriate.

^a^*p* < 0.05 compared with low PH probability group.

^b^*p* < 0.05 compared with intermediate PH probability group.

The 6MWD test showed different functional exercise capacity. The patients with intermediate PH probability and SSc-PH patients had shorter walking distances than the patients with low PH probability. The percentage of interstitial lung disease determined by HRCT was not significantly different among the groups. There was no difference in FE_NO_. The distribution of SSc subtypes and the use of corticosteroids, immunosuppressive agents, and vasodilator therapies were comparable across groups.

Pulmonary function test was performed before bronchodilator. Among the pulmonary function parameters, total lung capacity was lower in patients with SSc-PH than in the low probability group and the intermediate probability group. The diffusing capacity of the lungs for carbon monoxide was also lower in the SSc-PH group than in the low probability group.

### Endothelial function: Blood nitrite and FMD

**Fig 2:**
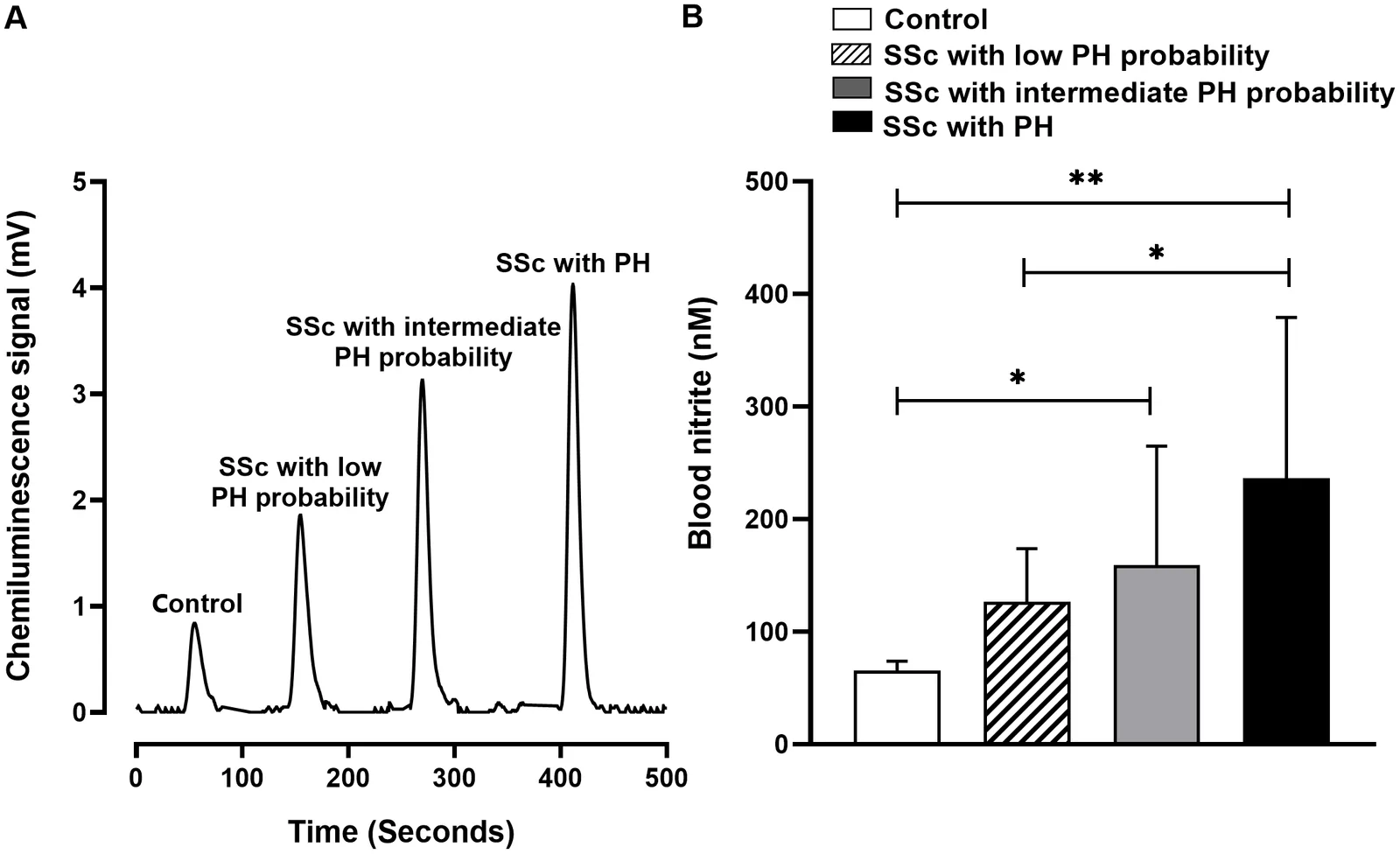
Blood nitrite levels in control and systemic sclerosis (SSc) patients. (A) Representative chemiluminescence traces obtained from blood nitrite measurements using tri-iodide–based reductive chemiluminescence in controls and SSc patients with low pulmonary hypertension (PH) probability, intermediate PH probability, and confirmed SSc-associated PH (SSc-PH). (B) Whole-blood nitrite concentrations in controls and SSc patients. Data are means ± standard deviation. *p < 0.05; **p < 0.001.

We measured blood nitrite and FMD, which represent endothelial function markers. The patients with PH and intermediate PH probability had higher blood nitrite levels than control (Fig 2). SSc-PH patients also had higher nitrite than the patients with low PH probability. The blood nitrite concentrations were 65.6 ± 8.1 nM in controls, 126.5 ± 47.3 nM in SSc patients with low PH probability, 158.9 ± 106.0 nM in SSc patients with intermediate probability, and 236.1 ± 143.0 nM in the SSc-PH patients. In contrast, plasma nitrate concentrations were not different among the groups (52.0 ± 16.2 μM in control, 71.2 ± 29.8 μM in SSc with low PH probability, 78.8 ± 37.2 μM in SSc with intermediate PH probability, and 77.6 ± 27.2 μM in SSc-PH).

**Fig 3:**
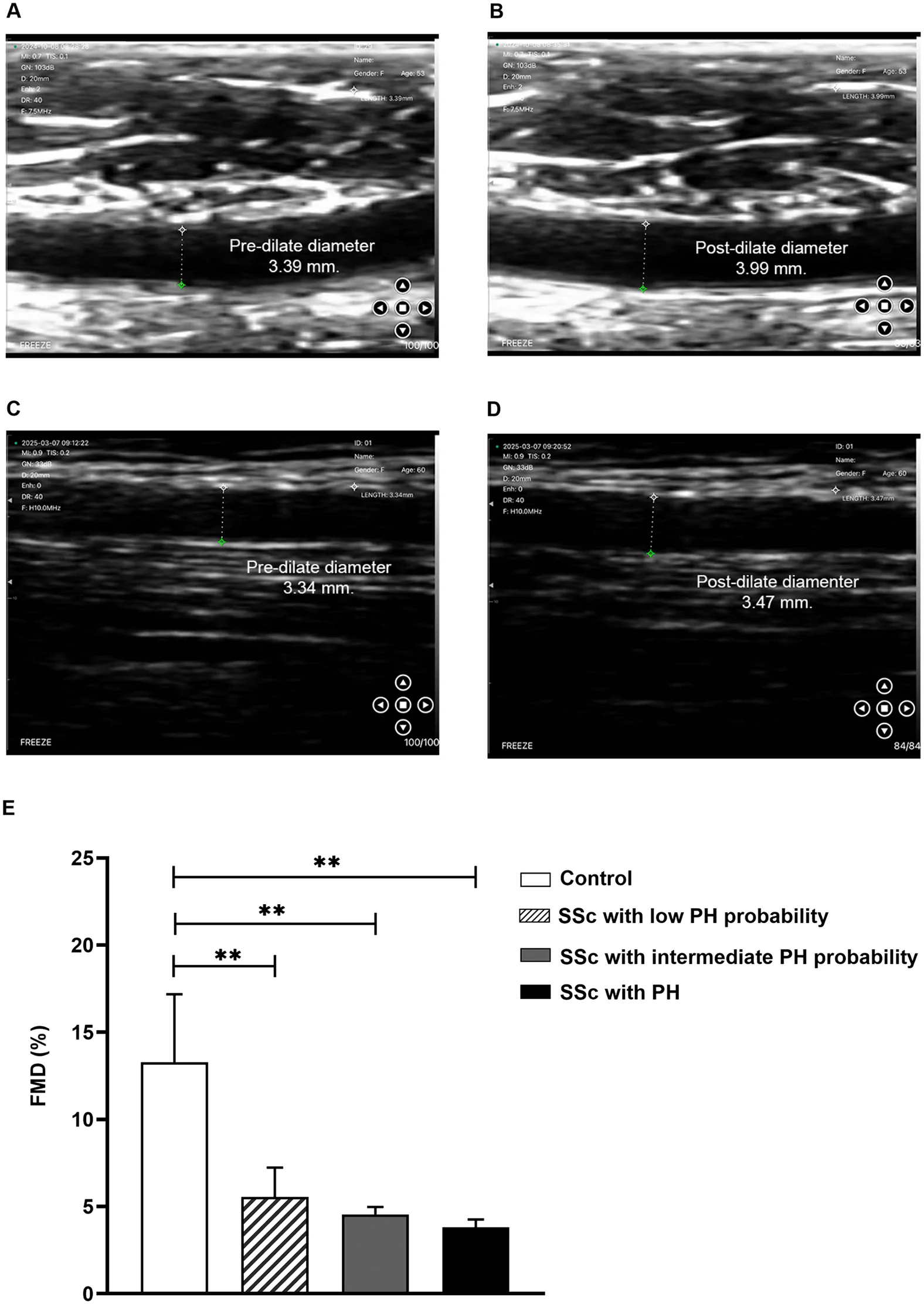
Flow-mediated dilation (FMD) of brachial artery. The ultrasound images of brachial artery of a healthy control participant showing vessel diameter before cuff inflation (A) and after reactive hyperemia (B), and of a patient with systemic sclerosis with pulmonary hypertension (SSc-PH) before cuff inflation (C) and after reactive hyperemia (D). Green calipers indicate arterial diameters. Comparison of FMD (%) in controls, systemic sclerosis (SSc) patients with low pulmonary hypertension (PH) probability, intermediate PH probability, and confirmed SSc-PH (E). Data are means ± standard deviation. **p < 0.001.

Baseline brachial artery diameter was comparable among the control, low PH probability, intermediate PH probability, and SSc-PH groups (3.14 ± 0.19, 3.15 ± 0.32, 3.07 ± 0.35, and 3.29 ± 0.06 mm, respectively; *p* = 0.40). Intra-observer reproducibility of FMD measurements was excellent, with a single-measure ICC of 0.984 (95% CI, 0.939–0.996; *p* < 0.001). All subgroups of SSc patients had lower FMD than control (Fig 3). The FMD change were 13.3 ± 3.9% in control, 5.6 ± 1.7% in the SSc with low PH probability, 4.6 ± 0.4% in SSc with intermediate PH probability, and 3.8 ± 0.5% in SSc-PH.

### Platelets

**Fig 4:**
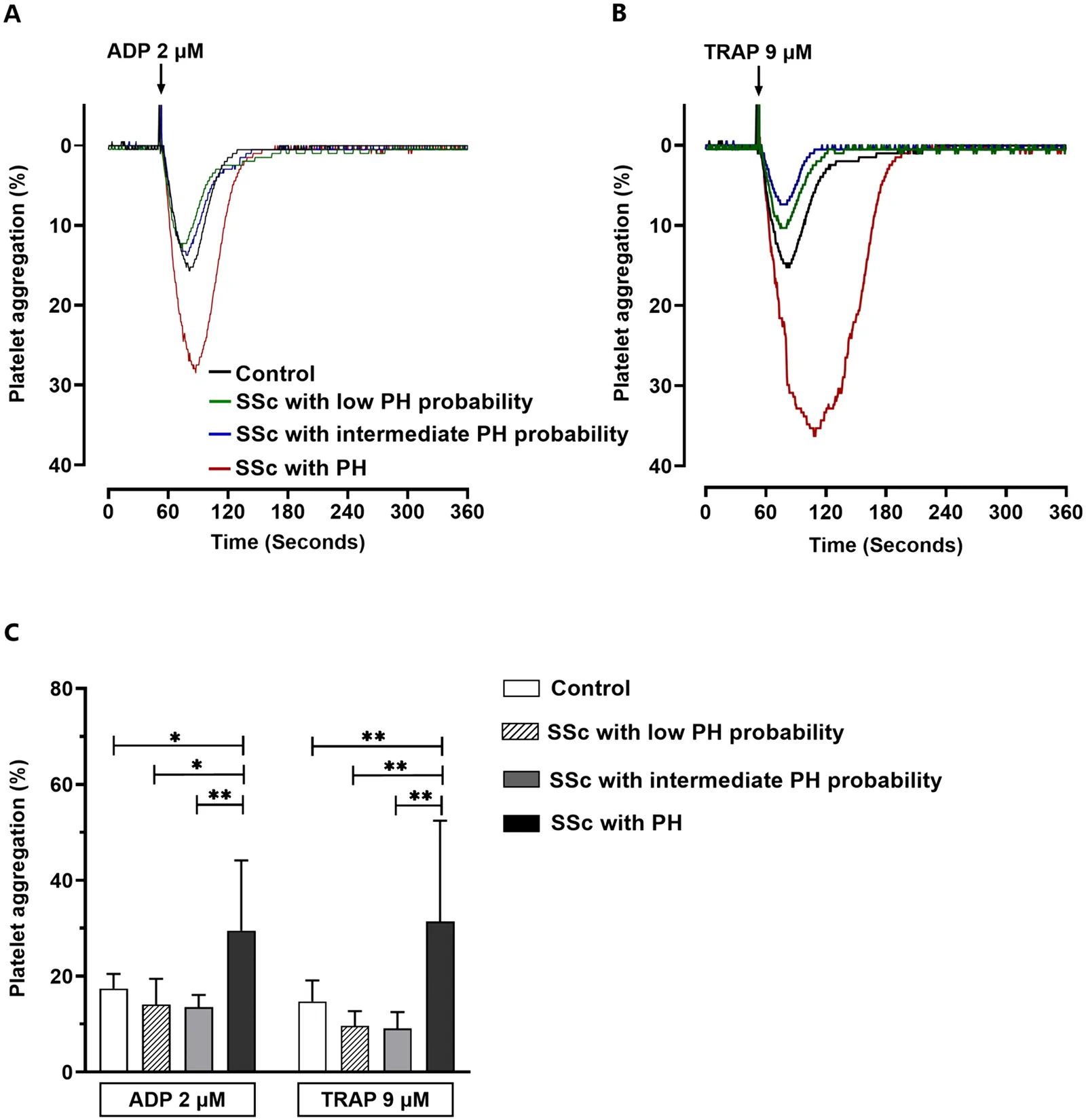
Platelet aggregation in response to stimulation by adenosine diphosphate (ADP) and thrombin receptor-activating peptide-6 (TRAP-6) in controls and systemic sclerosis (SSc) patients with low pulmonary hypertension (PH) probability, intermediate PH probability, and confirmed PH. (A) Representative platelet aggregation tracings after stimulation with ADP (2 μM) in control and SSc patients. (B) Representative aggregation tracings after stimulation with TRAP-6 (9 μM). (C) Maximal platelet aggregation induced by ADP (2 μM) and TRAP-6 (9 μM). Data are means ± standard deviation. *p < 0.05; **p < 0.001.

The maximal platelet aggregation in response to 2 μM ADP stimulation was higher in SSc-PH patients than in control and in patients with low and intermediate PH probability (Fig 4, S1 Table). Similarly, platelet aggregation induced by 9 μM TRAP-6 was significantly higher in the SSc-PH group than in control and patients with intermediate and low PH probability.

**Fig 5:**
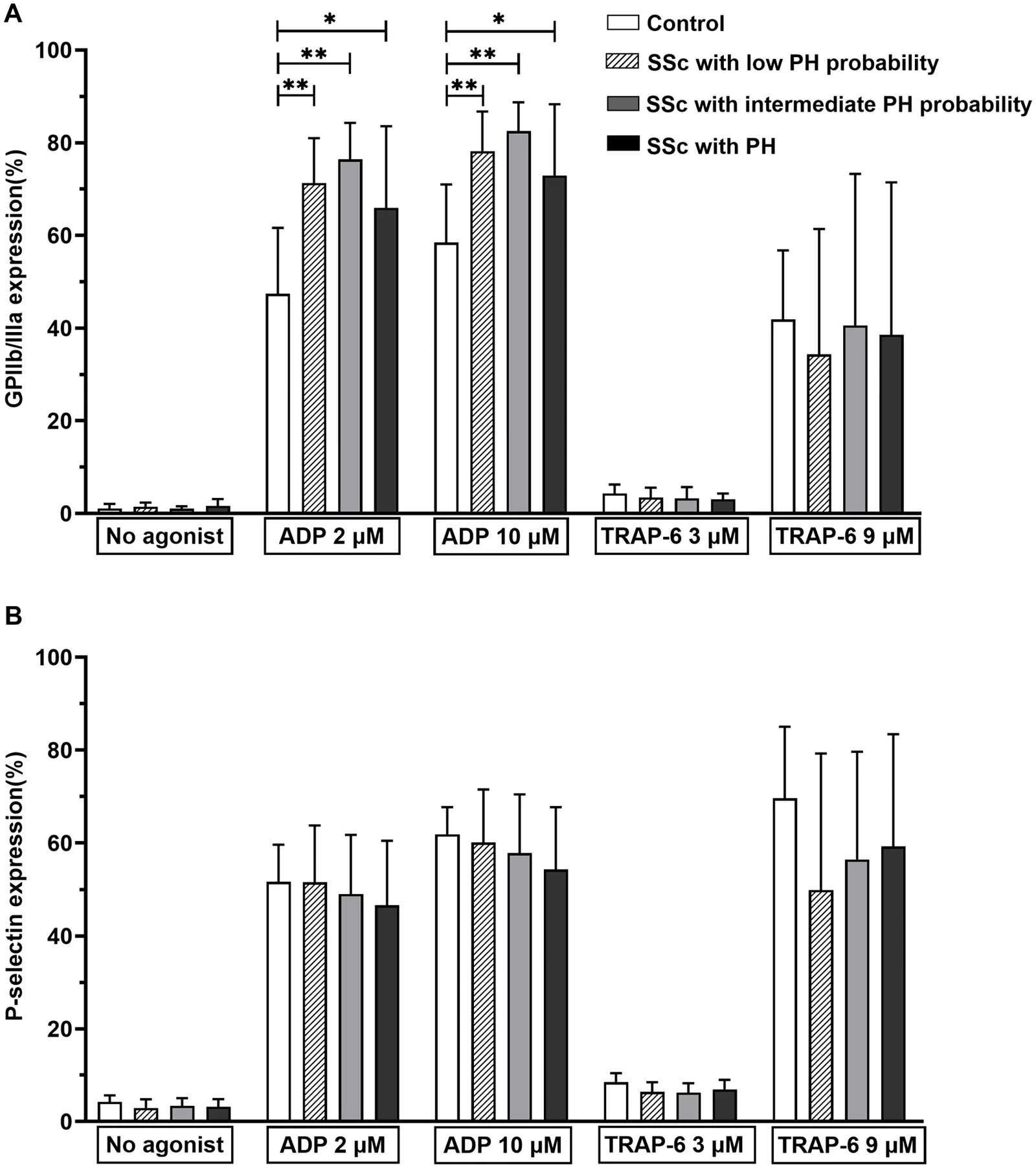
Platelet expression of glycoprotein (GP) IIb/IIIa (A) and P-selectin (B) in controls and SSc patients. Platelets were stimulated by adenosine diphosphate (ADP) or thrombin receptor-activating peptide-6 (TRAP-6) in controls and systemic sclerosis (SSc) patients with low pulmonary hypertension (PH) probability, intermediate PH probability, and confirmed PH. Data are means ± standard deviation. *p < 0.05; **p < 0.001.

By flow cytometry, GPIIb/IIIa expression in response to 2 and 10 μM ADP stimulation was higher in all SSc subgroups than in control subjects (Fig 5, S1 Table). No significant intergroup differences in GPIIb/IIIa expression were observed after TRAP-6 stimulation. P-selectin expression did not differ among groups at baseline and after stimulation with ADP or TRAP-6.

### Transthoracic echocardiography

The left-sided heart structural and functional parameters, including left atrial size, left ventricular dimensions, volumes, mass index, geometry, and ejection fraction, were comparable among the low-, intermediate-, and SSc-associated PH groups (Table 3). No significant difference was seen. Indices of left ventricular diastolic function and mitral inflow parameters were not different across groups.

**Table 3:** Echocardiographic data.

Parameters	SSc with low PH probability (n = 12)	SSc with intermediate PH probability (n = 7)	SSc-PH (n = 8)
**Left heart parameters**			
LA diameter, mm	33.83 ± 4.97	34.57 ± 5.74	30.61 ± 5.82
LA/Ao	1.20 ± 0.19	1.31 ± 0.32	1.30 ± 0.32
LA volume index, g/m^2^	26.37 ± 8.00	29.83 ± 8.68	31.15 ± 7.53
LV mass index, g/m^2^	83.39 ± 25.53	67.25 ± 5.30	1.1± 22.59
Abnormal LV geometry, n	4	2	5
LV ejection fraction, %	64.35 ± 7.96	68.39 ± 7.76	67.95 ± 5.49
LV systolic dysfunction, n	1	1	0
MV peak E velocity, m/s	0.75 ± 0.18	0.72 ± 0.18	0.70 ± 0.20
MV peak A velocity, m/s	0.72 (0.70–0.91)	0.75 (0.60–0.87)	0.87 (0.82–1.08)
MV peak E/A ratio	0.96 ± 0.29	0.97 ± 0.14	0.75 ± 0.16
MV deceleration time, s	0.21 ± 0.06	0.16 ± 0.03	0.23 ± 0.08
LV diastolic dysfunction, n	2	2	3
**Right heart parameters**			
RV TAPSE, mm	20.72 ± 2.75	20.31 ± 2.30	18.19 ± 3.33
RV lateral S’, cm/s	11.31 ± 0.88	9.99 ± 2.35	10.80 ± 1.82
RV systolic dysfunction, n	1	1	2
TAPSE/sPAP ratio	0.78 ± 0.21	0.53 ± 0.05 ^a^	0.31 ± 0.09 ^a,b^
**Pulmonary vasculature parameters**			
MPA diameter, mm	19.80 ± 1.83	20.00 ± 1.25	28.71 ± 4.63 ^a,b^
RAP, mmHg	8 (3-8)	8 (8−8)	8 (8-13.25)
TRV_max_, m/s	2.32 ± 0.24	2.71 ± 0.16 ^a^	3.50 ± 0.49 ^a,b^
RVSP, mmHg	28.38 ± 5.45	38.53 ± 3.30	58.53 ± 14.23 ^a,b^
mPAP (sys-dias), mmHg	16.54 ± 3.57	21.01 ± 2.62	31.59 ± 7.08 ^a,b^

A, late diastolic inflow velocity; E, early diastolic inflow velocity; LA, left atrium; LA/Ao, left atrial–to–aortic root diameter ratio; LV, left ventricle; MPA, mean pulmonary artery; mPAP, mean pulmonary artery pressure; MV, mitral valve; PH, pulmonary hypertension; RAP, right atrium pressure; RV, right ventricle; RVSP, right ventricular systolic pressure; sPAP, systolic pulmonary artery pressure; SSc, systemic sclerosis; TAPSE, tricuspid annular plane systolic excursion; TRV, tricuspid regurgitant velocity.

Values are presented as means ± standard deviation or medians (interquartile range), as appropriate.

^a^*p* < 0.05 compared with low PH probability group.

^b^*p* < 0.05 compared with intermediate PH probability group.

Within the right-sided heart parameters, SSc-PH patients had lower TAPSE/sPAP ratio than the patients with low and intermediate PH probability, indicating impaired RV–PA coupling. The TRV_max_, RV systolic pressure, and mPAP were significantly higher in patients with SSc-PH than in those with low or intermediate PH probability. Other RV systolic function parameters were not different across the groups.

### Correlation and regression analyses

Correlation and regression analyses were conducted to examine relationships between TRV_max_ with vascular, platelet, and functional variables. TRV_max_ displayed moderately inverse correlations with FMD and 6MWD (Table 4, S2 Fig). TRV_max_ also showed mildly-to-moderately positive correlations with ADP- and TRAP-6-induced platelet aggregation. TAPSE/sPAP ratio exhibited a positive correlation with FMD (*r* = 0.43, *p* = 0.023) and an inverse correlation with TRV_max_ (*r* = −0.66, *p* < 0.001). There was no correlation between TRV_max_ and blood nitrite.

**Table 4:** Spearman’s correlation.

Parameters	TRV_max_	
	*r*	*p*
FMD	−0.55	0.003
6MWD	−0.43	0.026
ADP-induced platelet aggregation	0.54	0.004
TRAP-6-induced platelet aggregation	0.45	0.018
Blood nitrite	0.34	0.081

6MWD, six-minute walk distance; ADP, adenosine diphosphate; FMD, flow-mediated dilatation; TRV, tricuspid regurgitant velocity; TRAP-6, thrombin receptor–activating peptide-6.

Spearman’s rank correlation coefficients (r) and *p*-values are shown.

Multiple linear regression using the enter method demonstrated that both TRAP-6-induced platelet aggregation (β = 0.476, *p* = 0.007) and FMD (β = −0.339, *p* = 0.045) independently associated with TRV_max_. The model explained 42.3% of the variance in TRV_max_ (R² = 0.426). Regression diagnostics showed no evidence of multicollinearity (all VIFs = 1.066), and the Durbin–Watson statistic was 1.996, indicating independence of residuals. Visual inspection of residual plots demonstrated no substantial deviation from normality or homoscedasticity. Bootstrap analysis using 1,000 resamples confirmed the stability of both regression coefficients, with BCa 95% confidence intervals excluding zero for FMD (−0.428 to −0.033) and TRAP-6-induced platelet aggregation (0.008 to 0.029) (S2 Table).

### Acute effects of inhaled sodium nitrite

**Fig 6:**
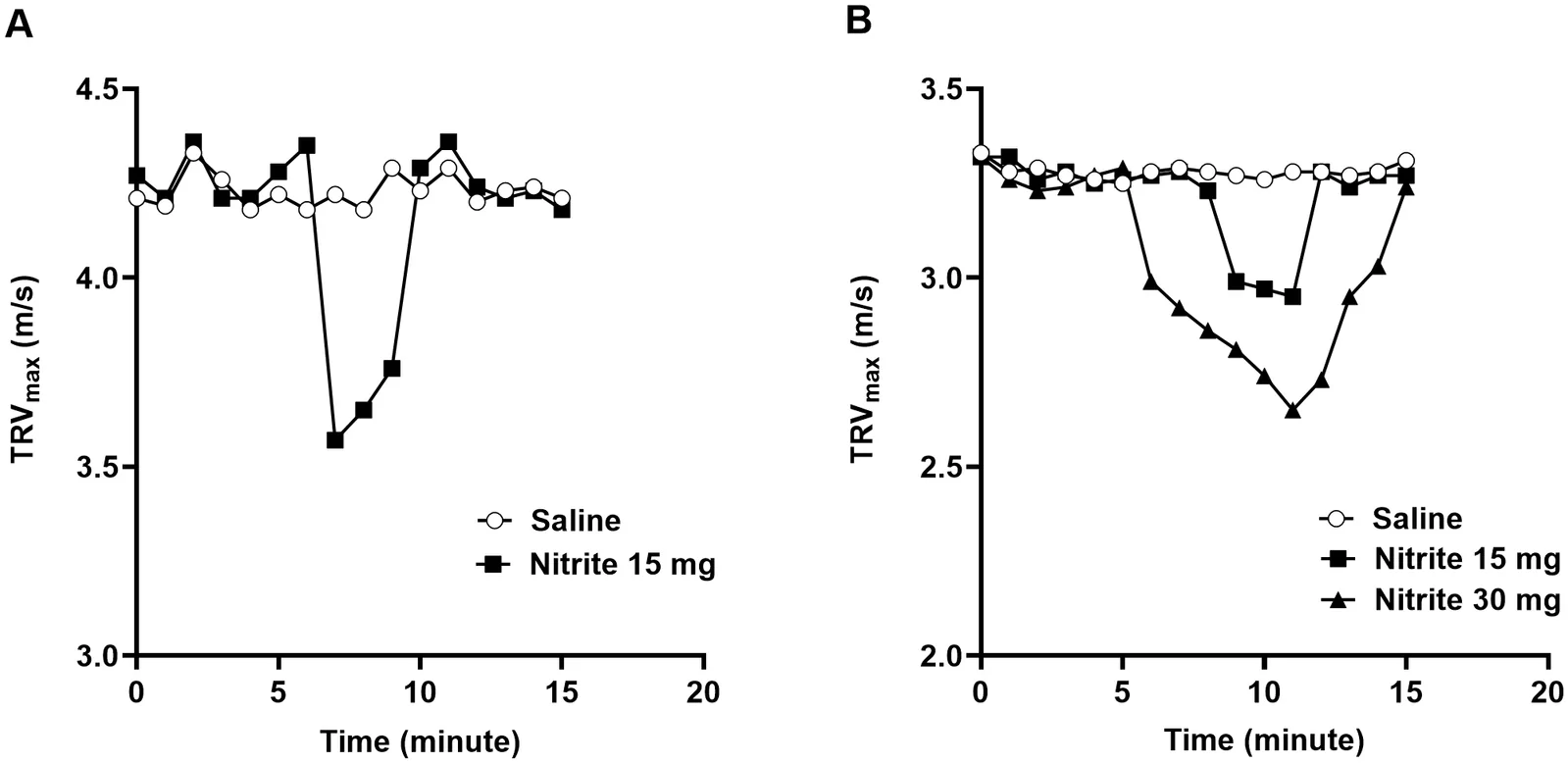
Effects of inhaled sodium nitrite on the maximal tricuspid regurgitant velocity (TRV_max_). The changes in TRV_max_ during sodium nitrite inhalation in patient 1 (systemic sclerosis with pulmonary hypertension) (A) and patient 2 (systemic sclerosis with intermediate probability of pulmonary hypertension) (B).

To investigate the acute hemodynamic effects of inhaled sodium nitrite in SSc, two patients were enrolled. Patient 1 (SSc-PH) had a transient decrease in TRV_max_ at the time 5–10 minutes of inhalation of 15-mg sodium nitrite (Fig 6A). The TRV_max_ decreased from 4.2 m/s at baseline to 3.6 m/s, representing an estimated reduction of 14%, and then gradually returned to baseline after inhalation. Systemic blood pressure and heart rate did not change. Blood nitrite increased from 157.3 nM at baseline to 240.3 nM immediately after nitrite inhalation. Blood nitrite declined to 217.8 nM and 200.3 nM at 15 and 30 minutes after inhalation, respectively.

In patient 2 (SSc with intermediate PH probability), there was a decrease in TRV_max_ with increasing doses of sodium nitrite (15 mg, then 30 mg) (Fig 6B). Inhalation of 15 mg sodium nitrite produced a slightly transient reduction in TRV_max_ from 3.3 m/s to 3.0 m/s (10% decrease). Subsequent inhalation of 30-mg sodium nitrite produced a more reduction, with TRV_max_ decreasing to 2.6 m/s (21% reduction), followed by a gradual recovery. Blood nitrite increased from 210.2 nM at baseline to 357.9 nM after the 15-mg dose, and then to 701.9 nM after the 30-mg dose, with gradual declines to 616.1 nM and 517.4 nM at 15- and 30-minutes after inhalation, respectively.

Blood pressure and heart rate were not changed (S3 Fig). In patient 1, methemoglobin levels remained stable: 8.6% at baseline and 8.5% at 15 minutes after inhalation. In patient 2, methemoglobin levels were 16.7% at baseline and 13.6% at 15 minutes after inhalation.

## Discussion

We integrated endothelial function testing, NO–related biomarkers, platelet reactivity, and echocardiographic assessment to evaluate vascular abnormalities across varying PH probabilities in SSc patients. A key finding is that endothelial dysfunction was evident in SSc patients with a low echocardiographic PH probability and became progressively more pronounced with increasing TRV_max_. FMD, which represents shear stress–induced endothelium-dependent vasodilation largely mediated by NO, declined in SSc patients. The inverse correlation between FMD and TRV_max_ suggests that worsening endothelial function would account, at least in part, for PH development. This finding supports the concept that endothelial dysfunction is a feature of pulmonary vascular disease in SSc.

The pattern observed for NO-related biomarkers provides important mechanistic insight into this endothelial dysfunction. Blood nitrite levels increased with higher PH probability despite impaired FMD. This apparent dissociation can be explained by altered NO handling in SSc. FMD reflects functional, shear stress–dependent endothelial NO signaling, whereas circulating nitrite represents a stable pool of NO metabolites derived from multiple sources, including inflammatory cells, and does not necessarily indicate effective NO bioavailability in the vascular wall [20].

Endothelial dysfunction in SSc is caused by locally impaired NO bioavailability at physiologic levels in paracrine manner, as reflected by decreased FMD. Oxidative stress, which promotes fibrosis in SSc [21], can induce tetrahydrobiopterin oxidation (eNOS cofactor) and eNOS protein thiyl radical formation, resulting in eNOS uncoupling [22,23]. In this uncoupled state, eNOS preferentially produces superoxide rather than NO, further reducing NO bioavailability and amplifying oxidative stress. Furthermore, SSc- PH has been related to elevated levels of asymmetric dimethylarginine, an endogenous eNOS inhibitor [24]. Excessive circulatory NO rapidly reacts with superoxide to produce peroxynitrite [25], which further promotes oxidative and nitrosative stress and impairs endothelial function. In this context, the elevated nitrite levels in our study may reflect pathologic NO production rather than physiologically endothelial NO signaling. The lack of a correlation between nitrite levels and TRV_max_ supports the concept that systemic nitrite levels do not represent functional endothelial NO bioavailability in this disease.

In this study, platelet aggregation increased in SSc-PH, as evidenced by the increased responses to both ADP and TRAP-6 stimulation. TRV_max_ exhibited positive correlations with ADP- and TRAP-induced aggregation, and TRAP-induced aggregation remained independently associated with TRV_max_ in multiple regression analysis. Platelet activation is physiologically suppressed by NO as a paracrine released by endothelial cells. Despite an increased in systemic nitrite, local NO bioavailability is insufficient as reflected by the reduced FMD, which is related with the increased responsiveness of platelets to agonists.

The relation of TRAP-6-induced aggregation and TRV_max_ implies that thrombin-mediated platelet signaling may be particularly relevant in the pulmonary vascular milieu of SSc-PH. Thrombin is a potent platelet agonist that activates platelets via protease-activated receptors, thereby amplifying platelet aggregation and promoting thrombo-inflammatory processes within the vascular wall, whereas ADP is a weak agonist and represents a secondary amplification pathway of platelet activation [26]. As platelets are produced in the lungs at substantial amount [27], compromised pulmonary function in PH can alter platelet functions through several mechanisms including chronic hypoxia, endothelial dysfunction (as seen in lower FE_NO_ in SSc-PH despite no statistical significance), increased mechanical shear stress from pulmonary fibrosis, and localized inflammation, altogether leading to platelet hyper-reactivity [28]. In pulmonary vascular disease, thrombin and protease-activated receptor signaling result in endothelial activation, smooth muscle proliferation, and coagulation. In addition, activated platelets release proliferative and vasoactive mediators, such as platelet-derived growth factor and transforming growth factor, which are increased in bronchoalveolar lavage fluid of SSc patients [29]. These mediators can induce vasoconstriction and smooth muscle proliferation – a characteristic of pulmonary vascular remodeling.

Endothelial dysfunction as shown by reduced FMD, altered NO metabolism, and platelet hyperreactivity are mechanistically linked components of the same vascular disturbance. Rather than isolated abnormalities, they appear as interconnected features of a dysregulated endothelial–platelet axis in SSc-PH. The parallel associations of reduced FMD and increased aggregation with higher TRV_max_ support the concept that endothelial dysfunction and platelet hyperreactivity evolve together as pulmonary vascular burden increases.

Echocardiography showed a gradual increase in TRV_max_, RVSP, and estimated mPAP across PH probability groups, indicating progressive pulmonary vascular loading. The left ventricular structure and systolic function were intact, indicating that increased pulmonary pressures were unlikely to result from left-sided heart disease and were more compatible with a pre-capillary pulmonary vascular phenotype. In addition to estimating pulmonary pressure, echocardiographic findings show RV adaptation to an increased afterload. The gradual decrease in the TAPSE/sPAP ratio among PH probability groups in our cohort provides significant insight into RV-PA coupling in SSc. TAPSE physiologically indicates longitudinal RV systolic shortening, while sPAP represents pulmonary afterload. Their ratio is used as a non-invasive surrogate for ventricular–arterial coupling, which is comparable to the invasive Ees/Ea ratio derived from pressure–volume analysis [8]. A preserved ratio indicates sufficient contractile adaptation to afterload, whereas a decreased ratio means inadequate RV contractile reserve in relation to pulmonary vascular load, indicating a change into maladaptive remodeling. In a previous large-scale analysis of 355 SSc patients, a TAPSE/sPAP ratio < 0.55 mm/mmHg was independently associated with PH and identified as a potential predictive marker of PH [30]. Moreover, a TAPSE/sPAP ratio ≤ 0.32 mm/mmHg was shown to predict all-cause mortality in SSc. The fact that patients in the intermediate-probability group in our study already exhibited a TAPSE/sPAP ratio near the previously established PH threshold suggests that RV–PA uncoupling may begin before overt hemodynamic PH is confirmed. Conversely, the markedly reduced ratio observed in SSc-PH patients aligns closely with mortality-associated cutoffs, indicating more advanced RV dysfunction in this cohort.

Physiologic NO release, as reflected by lower FMD, is impaired in SSc patients. To test whether local NO supplement could decrease pulmonary pressure, we tested the effect of inhaled sodium nitrite on TRV_max_. Inhaled sodium nitrite resulted in transient reductions in TRV_max_ without clinically significant systemic effects. These results are consistent with prior experimental and clinical studies demonstrating that inhaled nitrite therapy is safe and effective in reducing mPAP and pulmonary vascular resistance in adult patients with PH of diverse etiologies, including heart failure with preserved ejection fraction and thalassemia [17,31,32]. Nitrite serves as a significant reservoir for NO in the body, where it can be reduced to bioactive NO in pulmonary vasculature particularly under hypoxic and acidic conditions. This process is facilitated by various mechanisms involving deoxygenated hemoglobin, myoglobin, and xanthine oxidoreductase, and occurs independently of eNOS activity. Unlike inhaled NO gas, nitrite has a longer half-life and can be selectively converted to NO within the pulmonary circulation, making it a promising candidate for pulmonary vasodilator.

Our study has several limitations. As 80% of patients diagnosed with SSc are females and we could recruit only female patients, our data applies only with females. Its cross-sectional design limits causal inference, and invasive hemodynamic validation was not consistently performed. Additionally, the molecular mediators associated with oxidative stress and signaling in platelets were not determined. Future longitudinal research is needed to better understand how endothelial and platelet functions can aid in the early identification of individuals at risk for pulmonary hypertension. Furthermore, a detailed investigation into NO-based interventions may deepen our understanding of their clinical relevance in systemic sclerosis-associated pulmonary vascular pathology.

The other limitation of this study is the use of pulmonary vasodilator therapy in a subset of patients with SSc-PH. Phosphodiesterase 5 inhibitors and endothelin receptor antagonists have been shown to improve pulmonary hemodynamics [33,34]. Sildenafil, a phosphodiesterase 5 inhibitors, has also been reported to enhance endothelial function [35] and to potentiate NO-mediated inhibition of platelet aggregation in PH [36]. Nevertheless, patients with SSc-PH in our cohort continued to exhibit impaired endothelial function and enhanced platelet aggregation despite receiving background pulmonary vasodilator therapy. These findings suggest that endothelial dysfunction and platelet hyperreactivity persist despite current standard therapy, reflecting ongoing pulmonary vascular pathology in patients with established SSc-PH rather than being fully corrected by vasodilator treatment. Because pulmonary vasodilators were prescribed according to clinical indications and were used predominantly in patients with established SSc-PH, their independent effects could not be distinguished from disease severity in this relatively small cohort. Therefore, adjustment for vasodilator therapy or subgroup analysis would be highly susceptible to confounding by indication. Future studies including larger cohorts, particularly treatment-naïve patients, are warranted to clarify the independent effects of pulmonary vasodilator therapy on endothelial dysfunction and platelet activation.

## Conclusion

In SSc, FMD is decreased even in patients with low PH probabilities while the platelet reactivity is increased. TRV_max_ and TAPSE/sPAP ratio are association with FMD and platelet reactivity. Inhaled sodium nitrite acutely decreases TRV_max_.

## Supporting information

S1 Table Comparative platelet aggregation and activation responses across study groups. (DOCX)

S2 Table Prespecified multiple linear regression analysis of factors independently associated with TRV_max_ in systemic sclerosis patients. (DOCX)

S1 Fig Dose–response curves of adenosine diphosphate (ADP)- and thrombin receptor-activating peptide-6 (TRAP-6)-induced platelet aggregation in healthy volunteers used for agonist selection. Platelet aggregation was measured by the light transmission aggregometer with platelet-rich plasma obtained from healthy volunteers (n = 4). Aggregation was induced using increasing concentrations of ADP (A) and TRAP-6 (B). Platelet aggregation responses are presented as the area under the aggregation curve (AUC). Data are shown as means ± SD.
(TIF)

S2 Fig Correlation between the maximal tricuspid regurgitant velocity (TRV_max_) with flow-mediated dilation, ADP-induced platelet aggregation, thrombin receptor-activating peptide-6 (TRAP-6)-induced platelet aggregation, 6-min walk distance, and blood nitrite. (TIF)

S3 Fig Effects of inhaled sodium nitrite on systemic blood pressure. The systolic blood pressure (SBP), diastolic blood pressure (DBP), and mean arterial pressure (MAP) were recorded before, during (shade area) and after sodium nitrite inhalation in patient 1 (systemic sclerosis with pulmonary hypertension) (A) and patient 2 (systemic sclerosis with intermediate probability of pulmonary hypertension) (B).
(TIF)
