# Supplementary material for: Endothelial and platelet dysfunction in systemic sclerosis with pulmonary hypertension
Source: PLoS One. 2026 Sep 16;21(9):e0357987. doi: 10.1371/journal.pone.0357987 (PMC13581006; doi:10.1371/journal.pone.0357987)
Supplement: Supplementary file 1 [file pone.0357987.s001.docx]

Supplemental Table 1: Comparative platelet aggregation and activation responses across study groups.

| **Parameters** | **Control**  **(n=12)** | **SSc** | | |
| --- | --- | --- | --- | --- |
|  |  | **Low PH probability**  **(n=12)** | **Intermediate PH probability**  **(n=7)** | **Confirmed PH**  **(n=8)** |
| **Platelet aggregation (%)** |  |  |  |  |
| **ADP stimulation** |  |  |  |  |
| - 2 μM | 17.4 ± 3.1 | 14.1 ± 5.4 | 13.6 ± 2.5 | 29.5 ± 14.7 ^a, b,c^ |
| - 10 μM | 56.6 ± 7.1 | 53.4 ± 5.5 | 48.1 ± 8.8 | 53.9 ± 7.7 |
| **TRAP-6 stimulation** |  |  |  |  |
| - 3 μM | 4.2 ± 1.6 | 3.7 ± 1.4 | 3.4 ± 1.0 | 4.3 ± 2.1 |
| - 9 μM | 14.8 ± 4.4 | 9.7 ± 3.1 | 9.1 ± 3.4 | 31.4 ± 21.0 ^a,b,c^ |
| **GPIIbIIIa expression (%)** |  |  |  |  |
| **Baseline** | 1.1 ± 1.0 | 1.5. ± 0.9 | 1.0 ± 0.5 | 1.6 ± 1.5 |
| **ADP stimulation** |  |  |  |  |
| - 2 μM | 47.4 ± 14.2 | 71.3 ± 9.7^a^ | 76.4 ± 7.9 ^a^ | 65.9 ± 17.6 ^a^ |
| - 10 μM | 58.5 ± 12.5 | 78.2 ± 8.6 ^a^ | 82.5 ± 6.2 ^a^ | 72.9 ± 15.4 ^a^ |
| **TRAP-6 stimulation** |  |  |  |  |
| - 3 μM | 4.3 ± 2.0 | 3.4. ± 2.1 | 3.3 ± 2.4 | 3.1 ± 1.2 |
| - 9 μM | 41.8 ± 14.9 | 34.3 ± 27.0 | 40.6 ± 32.7 | 38.5 ± 32.9 |
| **P-selectin expression (%)** |  |  |  |  |
| **Baseline** | 4.3 ± 1.4 | 3.0 ± 1.9 | 3.4 ± 1.6 | 3.2 ± 1.7 |
| **ADP stimulation** |  |  |  |  |
| - 2 μM | 51.6 ± 8.0 | 51.5 ± 12.2 | 49.0 ± 12.7 | 46.6 ± 13.9 |
| - 10 μM | 61.9 ± 5.8 | 60.1 ± 11.4 | 57.8 ± 12.7 | 54.3 ± 13.4 |
| **TRAP-6 stimulation** |  |  |  |  |
| - 3 μM | 8.5 ± 1.9 | 6.4 ± 2.1 | 6.2 ± 2.1 | 6.9 ± 2.1 |
| - 9 μM | 69.6 ± 15.4 | 56.5 ± 23.2 | 49.9 ± 29.4 | 59.3 ± 24.2 |

Values are presented as mean ± standard deviation

^a^ *p* < 0.05 vs. control group; ^b^ *p* < 0.05 vs. low PH probability group, ^c^ *p* < 0.05 vs. intermediate PH probability group (one-way ANOVA with Tukey’s post hoc test)

Abbreviations: ADP, adenosine diphosphate; GP, glycoprotein; TRAP, thrombin receptor–activating peptide; SSc, systemic sclerosis; PH, pulmonary hypertension.
