# Supplementary material for: Endothelial and platelet dysfunction in systemic sclerosis with pulmonary hypertension
Source: PLoS One. 2026 Sep 16;21(9):e0357987. doi: 10.1371/journal.pone.0357987 (PMC13581006; doi:10.1371/journal.pone.0357987)
Supplement: Supplementary file 2 [file pone.0357987.s002.docx]

**S2 Table. Prespecified multiple linear regression analysis of factors independently associated with TRV_max_ in systemic sclerosis patients.**

| **Variable** | β | B (95% CI) | *p* value | Bootstrap BCa 95% CI for B |
| --- | --- | --- | --- | --- |
| FMD | −0.34 | −0.146 (−0.288 to −0.004) | 0.045 | −0.428 to −0.033 |
| TRAP-6-induced platelet aggregation | 0.48 | 0.018 (0.006 to 0.031) | 0.007 | 0.008 to 0.029 |

Variables were entered simultaneously using the enter method. Bootstrap estimates were generated from 1,000 resamples with BCa 95% confidence intervals. Model performance: R² = 0.426, adjusted R² = 0.375; Durbin–Watson statistic = 1.996; all VIFs = 1.066.

**Abbreviations:** BCa, bias-corrected and accelerated; CI, confidence interval; FMD, flow-mediated dilation; TRAP, thrombin receptor-activating peptide-6; TRV_max_, maximum tricuspid regurgitation velocity; VIF, variance inflation factor.
